# Cancer spreading patterns based on epithelial-mesenchymal plasticity

**DOI:** 10.3389/fcell.2024.1259953

**Published:** 2024-04-11

**Authors:** Rui Wang, Zhaopeng Yan

**Affiliations:** ^1^ Department of Critical Care Medicine, Shengjing Hospital of China Medical University, Shenyang, China; ^2^ Department of General Surgery, Shengjing Hospital of China Medical University, Shenyang, China

**Keywords:** metastasis, epithelial-mesenchymal transition, mesenchymal-epithelial plasticity, hybrid epithelial/mesenchymal phenotype, hysteresis

## Abstract

**Introduction:** Metastasis is a major cause of cancer-related deaths, underscoring the necessity to discern the rules and patterns of cancer cell spreading. Epithelial-mesenchymal plasticity contributes to cancer aggressiveness and metastasis. Despite establishing key determinants of cancer aggressiveness and metastatic ability, a comprehensive understanding of the underlying mechanism is unknown. We aimed to propose a classification system for cancer cells based on epithelial-mesenchymal plasticity, focusing on hysteresis of the epithelial-mesenchymal transition and the hybrid epithelial/mesenchymal phenotype.

**Methods:** We extensively reviewed the concept of epithelial-mesenchymal plasticity, specifically considering the hysteresis of the epithelial-mesenchymal transition and the hybrid epithelial/mesenchymal phenotype.

**Results:** In this review and hypothesis article, based on epithelial-mesenchymal plasticity, especially the hysteresis of epithelial-mesenchymal transition and the hybrid epithelial/mesenchymal phenotype, we proposed a classification of cancer cells, indicating that cancer cells with epithelial-mesenchymal plasticity potential could be classified into four types: irreversible hysteresis, weak hysteresis, strong hysteresis, and hybrid epithelial/mesenchymal phenotype. These four types of cancer cells had varied biology, spreading features, and prognoses.

**Discussion:** Our results highlight that the proposed classification system offers insights into the diverse behaviors of cancer cells, providing implications for cancer aggressiveness and metastasis.

## 1 Introduction

Metastasis treatment is challenging and is the leading cause of cancer-related mortality (Mehlen and Puisieux, 2006; Chaffer and Weinberg, 2011). Metastasis is a multistep process involving cancer cell migration and subsequent colonization. However, tumors are heterogeneous, indicating that even in the same tumor, cancer cells exhibit variant biological behaviors and corresponding aggressiveness. Since cancer cells exhibit various proliferative, migratory, and colonization abilities, metastasis is unpredictable and complicated. Therefore, elucidating the rules and patterns governing cancer cell spread holds significant clinical implications.

Metastasis is closely associated with the epithelial-mesenchymal transition (EMT). The EMT allows epithelial cancer cells to acquire mesenchymal properties and subsequently gain migratory abilities (Batlle et al., 2000; Cano et al., 2000). Mesenchymal-epithelial transition (MET) is the process by which epithelial cancer cells transition to the epithelial state and regain their proliferative ability. Epithelial-mesenchymal plasticity (EMP) allows cancer cells to interconvert between multiple states across the epithelial-mesenchymal spectrum, contributing to cancer cell migration and subsequent colonization. Hysteresis of EMT refers to the maintenance of the mesenchymal state in mesenchymal cancer cells even after exiting the microenvironment, which induces EMT (Berenguer and Celià-Terrassa, 2021). It is associated with aggressive cancer and poor prognosis (Berenguer and Celià-Terrassa, 2021). The hybrid epithelial/mesenchymal phenotype is characterized by cancer cells maintaining both traits of epithelial and mesenchymal cancer cells. This hybrid state of cancer cells is highly aggressive and is associated with cancer stemness (Lambert and Weinberg, 2021a).

Based on EMP, EMT hysteresis, and the hybrid epithelial/mesenchymal phenotype, we propose a classification for cancer cells with EMP potential. This classification aims to interpret the behavior of cancer cells during the spreading process. This classification divides cancer cells with EMP potential into four types: 1) irreversible hysteresis-type cancer cells can cause stochastic metastasis, expressing an oligometastatic and metachronous metastatic pattern; 2) weak hysteresis-type cancer cells can cause distal metastases with low efficacy; 3) strong hysteresis-type cancer cells acquire a temporary hybrid epithelial/mesenchymal phenotype and effectively metastasize; and 4) stable hybrid epithelial/mesenchymal phenotype cancer cells are highly aggressive and can cause rapid and wide metastases.

## 2 Epithelial-mesenchymal plasticity

Epithelial-mesenchymal transition (EMT) and its reverse mesenchymal-epithelial transition (MET) are critical during embryonic development, wound healing, tissue homeostasis, and cancer metastasis (Ahmed et al., 2006; Nieto et al., 2016; Serrano-Gomez et al., 2016; Dongre and Weinberg, 2019). During EMT, non-motile epithelial cells transdifferentiate into mesenchymal cells, losing cell polarity and adhesion to acquire migratory properties. Further, EMT plays a critical role in promoting metastasis of epithelium-derived carcinomas (Batlle et al., 2000; Cano et al., 2000; Tsai and Yang, 2013) and therapeutic resistance (Dong et al., 2021).

EMT-inducing signals from the tumor microenvironment (TME) can promote EMT. Stromal cells such as tumor-infiltrating lymphocytes, cancer-associated fibroblasts, tumor-associated macrophages, and myeloid-derived suppressor cells in the TME can induce EMT (Liu et al., 2013; Marvel and Gabrilovich, 2015; Hu et al., 2019; Salazar et al., 2020). The factors secreted by stromal cells or cancer cells that can promote EMT include TGF-β (Potts and Runyan, 1989; Miettinen et al., 1994), HGF (Liu et al., 2017), FGF (Vallés et al., 1990), EGF (Sheng et al., 2020), Wnt (Kim et al., 2002) and Notch (Timmerman et al., 2004). Other microenvironmental parameters such as hypoxia (Lester et al., 2007; Yang et al., 2008), deficiency in nutrients (Recouvreux et al., 2020; Nakasuka et al., 2021), shear forces (Heise et al., 2011; Przybyla et al., 2016; Zhao et al., 2021), and matrix rigidity (Fattet et al., 2020; Deng et al., 2021) also contribute to EMT-inducing. Besides factors from the TME, genetic alterations inside cancer cells can also induce EMT (Yan et al., 2014; Shi et al., 2022).

Mesenchymal cancer cells lack proliferative abilities and cannot successfully initiate metastasis. Further, MET is required for successful metastatic colonization (Chaffer et al., 2006; Korpal et al., 2011; Celià-Terrassa et al., 2012; Gunasinghe et al., 2012; Ocaña et al., 2012; Tsai et al., 2012; Tran et al., 2014; Del Pozo Martin et al., 2015; Beerling et al., 2016). The mesenchymal phenotype of cancer cells can be restored by undergoing MET (Kalluri and Weinberg, 2009; Nieto et al., 2016; Yang et al., 2020). Multiple factors regulate MET: 1) After migration out of the TME, withdrawal of EMT-inducing signals drives cancer cells to undergo MET (Eckley et al., 2019; Li et al., 2020; Berenguer and Celià-Terrassa, 2021). 2) Newly emerging genetic alterations that interfere with EMT-TFs lead to MET (Roca et al., 2013). 3) Noise or oscillations may drive the stochastic MET (Nordick et al., 2022).

Epithelial-mesenchymal plasticity refers to changes in the phenotype within the epithelial-mesenchymal spectrum. It reflects the ability of cancer cells to transition from an entirely epithelial to a mesenchymal state. Successful metastasis requires the proliferative ability of epithelial cancer cells and the migratory ability of mesenchymal cancer cells. Instead of EMT, EMPs contribute to tumor progression by promoting therapy resistance and immune cell evasion (Cook and Vanderhyden, 2022). Epithelial-mesenchymal plasticity is characterized by five distinct factors: local microenvironment, lineage specification, cell identity, genome, hysteresis (cell memory), and noise-driven stochastic state transitions (Cook and Vanderhyden, 2020; Haerinck et al., 2023).

## 3 The hysteresis of EMT

The term hysteresis describes the phenomenon in which the state of a system depends on its history. The epithelial-mesenchymal transition, a hysteresis process (Berenguer and Celià-Terrassa, 2021; Haerinck et al., 2023), implies that mesenchymal cancer cells can maintain their mesenchymal phenotype even after the withdrawal of EMT-inducing signals (Jia W. et al., 2019; Eckley et al., 2019; Li et al., 2020). For example, exposing cells to varying durations of TGF-β revealed that cells exposed for a shorter duration (3–6 days) reverted to being epithelial in a similar time frame. However, cells exposed for a longer duration (12–15 days) underwent a stronger degree of EMT, and not all of them reverted to being epithelial even after 15 days post-TGF-β removal (Jia W. et al., 2019).

The hysteresis phenomenon can be explained by: 1) the transition from the epithelial to the mesenchymal state requiring greater stimulus than that needed to maintain the mesenchymal state; 2) the potential perpetuation of mesenchymal state through robust positive or double-negative feedback loops, allowing cells to persist even after the complete withdrawal of the initial stimulus (Ferrell, 2002; Haerinck et al., 2023); and 3) incomplete gene expression level restoration upon reversal of EMT, creating a “transcriptional memory” (Stylianou et al., 2019).

Further, EMT hysteresis has prognostic value (Celià-Terrassa et al., 2018). The hysteresis control of EMT dynamics involves a distinct program with enhanced metastatic abilities (Celià-Terrassa et al., 2018). Both hysteretic and non-hysteretic EMT confer similar morphological changes and invasive potential to cancer cells; only hysteretic EMT enhances metastatic colonization efficiency. Cells that undergo hysteretic EMT differentially express subsets of stem cell- and extracellular matrix-related genes with significant clinical prognostic value (Celià-Terrassa et al., 2018).

The regulation mechanisms of hysteresis include: 1) Determination by lineage specification and cell identity, showcasing varied hysteresis abilities among different cancer cells (Schmidt et al., 2015). 2) Influence from the TME, where collagen organization, reflecting matrix stiffness, plays a key role. Matrix rigidity can trigger EMT, and cancer cells can remain in mesenchymal state for several days after leaving the microenvironment that induced EMT (Nasrollahi et al., 2017). 3) Epigenetic changes with chronic inflammation induced by IL-1β can regulate EMT memory phenotypes through epigenetic modifications (Li et al., 2020). Epigenetic memory acquired during prolonged EMT induction governs the recovery to the epithelial state (Jain et al., 2023). 4) Impact of transcription factors, where high expression of the transcription factor SLUG is essential for establishing EMT memory (Li et al., 2020).

Hysteresis emerges as a prevalent phenomenon in EMT. Cancer cells exhibit diverse hysteresis strengths, allowing them to remain in a mesenchymal state for various durations. The strength of the hysteresis is determined by lineage specifications, cell identity, stimulation intensity, and time from the TME. We artificially classified EMT hysteresis as irreversible, weak, or strong.

## 4 Hybrid epithelial/mesenchymal phenotype

### 4.1 Hybrid epithelial/mesenchymal phenotype has been proven at clinical and preclinical levels

A hybrid epithelial-mesenchymal phenotype has been proposed (Lambert and Weinberg, 2021b). Intermediate states between the epithelial and mesenchymal states have been identified as hybrid epithelial/mesenchymal states. This unique state does not represent a mixture of epithelial and mesenchymal cancer cells; however, cancer cells persist in a stable semi-epithelial and semi-mesenchymal state. They express both epithelial and mesenchymal markers while simultaneously exhibiting properties of both phenotypes (Kröger et al., 2019; Sinha et al., 2020).

The hybrid epithelial-mesenchymal phenotype has been demonstrated using multiple experimental and computational analyses at the cellular level (Zhang et al., 2014; Grosse-Wilde et al., 2015; Hari et al., 2020; Brown et al., 2022). At the animal level, recent lineage-tracing experiments in mouse models of metastatic breast cancer have suggested that a complete transition to a mesenchymal state is rare. However, cells often undergo partial EMT, leading to a hybrid epithelial/mesenchymal phenotype (Lüönd et al., 2021). Recent single-cell RNA (Puram et al., 2017; Dong et al., 2018; Pastushenko et al., 2018; McFaline-Figueroa et al., 2019; Cook and Vanderhyden, 2020; Ji et al., 2020; Zhao et al., 2020; Deshmukh et al., 2021; Simeonov et al., 2021) and protein (Pastushenko et al., 2018; Karacosta et al., 2019; Taverna et al., 2020) profiling has demonstrated that cells can linger in a series of intermediate states along the epithelial–mesenchymal axis (Pastushenko and Blanpain, 2019). At the clinical level, a hybrid phenotype has been demonstrated in cancers (Grosse-Wilde et al., 2015; Capellero et al., 2022).

### 4.2 Hybrid phenotype: more aggressive and poor prognosis

Compared with complete epithelial or mesenchymal cancer cells, hybrid-state cancer cells can simultaneously proliferate and migrate, possess stemness traits, and are more aggressive (Pastushenko et al., 2018; Kröger et al., 2019). Hybrid-state cancer cells are more metastatic than mesenchymal cancer cells in tumor initiation (Pastushenko et al., 2018; Kröger et al., 2019). *In vitro* observations revealed that hybrid E/M cells have an almost ten times higher mammosphere-forming ability than epithelial or mesenchymal cells (Grosse-Wilde et al., 2015). Hybrid epithelial and mesenchymal cancer cells also contribute to chemoresistance (Lüönd et al., 2021).

The hybrid epithelial/mesenchymal phenotype cancer cells exhibit high tumorigenic properties (Sinha et al., 2020), leading to stemness (Godin et al., 2020), metastasis (Bocci et al., 2017), therapy resistance (Sinha et al., 2020), and poor prognosis (Lee et al., 2014; Bocci et al., 2019a; Bocci et al., 2019b; Tripathi et al., 2020; Vilchez Mercedes et al., 2022). Acquiring a hybrid E/M state is essential for the tumorigenicity of basal breast cancer cells (Kröger et al., 2019).

Clinically, an association between hybrid phenotypes and poor prognosis has been proven. The co-expression of epithelial and mesenchymal signatures was associated with worse patient outcomes in luminal and basal breast cancers, highlighting the aggressive behavior of hybrid E/M cells (Grosse-Wilde et al., 2015). Recent sequential immunohistochemical analysis demonstrated that the presence of hybrid E/M cells was strongly associated with poor prognosis. Further, the presence of a minimum percentage of tumor cells in the hybrid E/M state (E/M score <2%) was sufficient to confer poor overall and disease-free survival in patients (Godin et al., 2020). This strong association between hybrid E/M cells and poor patient survival can be partly attributed to their enhanced stemness, drug resistance, and immune evasion.

### 4.3 The regulation of hybrid epithelial/mesenchymal phenotype

Multiple mechanisms contribute to a stable hybrid epithelial/mesenchymal phenotype governed by phenotypic stability factors, TFs, adherent junction proteins (E-cad and N-cad), epigenetic regulators, post-translational modifications, and the TME (Sinha et al., 2020).

A hybrid epithelial/mesenchymal phenotype arises from a balance between factors that either promote or inhibit EMT. Various contributors govern the origin and sustainability of this hybrid state: 1) A single factor contributes to the origin and maintenance of a hybrid phenotype. For example, NRF2 inhibits complete EMT and promotes a hybrid phenotype (Bocci et al., 2019b; Vilchez Mercedes et al., 2022). Numb acts as a brake for full EMT by modulating Notch-driven EMT and can inhibit full EMT and stabilize a hybrid phenotype. The knockdown of Numb in stable hybrid phenotype cells resulted in full EMT (Bocci et al., 2017). Transcriptional activation of Snai1 by Wilms’ tumor transcription factor (WT1) prevents the repression of E-cadherin and confers a hybrid E/M state (Sampson et al., 2014). Loss-of-function of cadherin Fat1 via mutation or deletion in mouse and human squamous cell carcinoma stimulates the mesenchymal state and sustains the epithelial state simultaneously. The integrated effect of the loss of function of cadherin Fat1 promotes a hybrid phenotype (Pastushenko et al., 2021). GRHL2 couples with the core EMT decision-making circuit (miR-200/ZEB) to stabilize the hybrid E/M phenotype (Jolly et al., 2016; Chung et al., 2019). YBX1 overexpression induces partial EMT by increasing the expression of several EMT-TFs (Gopal et al., 2015). Overexpression of HOTAIR lncRNA can maintain a hybrid phenotype (Topel et al., 2020). 2) Tumor microenvironment can contribute to a hybrid phenotype. For example, autocrine or paracrine effects of inflammatory cytokines in the TME, such as IL-6, can upregulate Notch-Jagged signaling, the activation of which can stabilize cells in hybrid E/M phenotypes (Bocci et al., 2019a). 3) Incomplete cellular reprogramming can lead to a hybrid E/M phenotype (Hiew et al., 2018; Jia D. et al., 2019). 4) Crosstalk between EMT feedback loops can contribute to the hybrid phenotype. For example, crosstalk between EMT feedback loops involving ESRP1, HAS2, and CD44 can enable cells to maintain a hybrid E/M phenotype (Jolly et al., 2018). 5) Epigenetic regulations. For example, GRHL2 is associated with the epigenetic regulation of hybrid EMT (Chung et al., 2019).

In addition to being stable, the hybrid phenotype can be transient or unstable (Biswas et al., 2019). We propose that cancer cells with substantial hysteresis in MET will not transition to the epithelial state instantaneously but will take some time. In this time-consuming MET process, cancer cells temporarily express both E-cadherin and M-cadherin and simultaneously possess epithelial and mesenchymal traits. We nominated this hybrid phenotype as a “temporary hybrid epithelial/mesenchymal phenotype.”

### 4.4 Conclusion of hybrid epithelial/mesenchymal phenotype

The hybrid epithelial/mesenchymal phenotype is common in cancers. Hybrid cancer cells are more aggressive and associated with a poor prognosis. There are two types of hybrid epithelial/mesenchymal phenotypes: stable and temporary. The temporary hybrid phenotype, although more prevalent, proved to be unstable and less aggressive than its stable counterpart.

## 5 Classification of cancer cells with EMT potential

Metastasis is a major cause of cancer-related deaths. Only a minute fraction of circulating tumor cells (<0.05%) survive and initiate metastasis (Abati and Liotta, 1996). Epithelial-mesenchymal plasticity contributes to tumor migration and metastasis. Therefore, in this study, we focused only on cancer cells with EMP potential instead of the entire tumor bulk. The heterogeneity of cancer cells determines the hysteresis of EMT variants. Cancer cells are classified into opportunistic irreversible, weak, and strong hysteresis. Additionally, hybrid epithelial/mesenchymal phenotype contributes to tumor aggressiveness. Based on EMP, especially the hysteresis of EMT and the hybrid epithelial/mesenchymal state, we propose that cancer cells with EMP can be classified into four types: opportunistic irreversible hysteresis, weak hysteresis, strong hysteresis, and stable hybrid phenotype.

### 5.1 Type-1 (opportunistic irreversible hysteresis type)

Genetic or epigenetic alterations inside cancer cells can induce EMT, and as the EMT-inducing signals persist from the inside, they can sustain cancer cells in a mesenchymal state, even in the left TME. Sometimes, signals from the TME can induce irreversible EMT (Jia W. et al., 2019). These stable mesenchymal cancer cells cannot transition back to the epithelial state spontaneously; we nominate this scenario as “irreversible hysteresis.” However, this irreversible mesenchymal phenotype is not absolute. Later, stochastic MET-inducing factors emerged in the TME of the target organ, stochastic genetic or epigenetic alterations inside cancer cells, or microRNA-mRNA interaction-driven oscillations; MET can be activated, and these mesenchymal cancer cells can regain the epithelial phenotype and, correspondingly, regain epithelial traits, such as proliferation. We nominate the MET of this scenario as “opportunistic.”

According to this definition, we speculate that Type-1 cancer cells exhibit distinctive traits: Firstly, they demonstrate low plasticity, marked by an “irreversibility” that restricts the free transition between mesenchymal and epithelial states. This limited plasticity is a defining feature of these cells. Secondly, Type-1 cancer cells display less aggressiveness and relatively favorable prognosis compared to other types but inferior to tumors lacking cancer cells with EMT potential (Eichelberger et al., 2020). Thirdly, the complete mesenchymal state in Type-1 cancer cells, characterized by a lack of E-cadherin, prompts individual migration—specifically, single-cell migration—rather than the collective migration seen in clustered cells.

### 5.2 Type-2 (weak hysteresis type)

The TME often plays a crucial role in promoting EMT. Tumor stromal cells in TME, such as TILs, could secrete EMT-inducing factors (for example, TGF-β) to induce cancer cells EMT. After EMT, migratory mesenchymal cancer cells exit the TME of the primary lesion. The cessation of EMT-inducing factors prompts a spontaneous MET. Cancer cells with weak hysteresis, capable of sustaining in a mesenchymal state for a limited period, migrate a constrained distance, causing limited spread. Subsequently, they transition back to the epithelial state, restoring traits associated with the epithelial phenotype, such as adhesion and proliferation. We categorize cancer cells undergoing EMT driven by TME with weak hysteresis as “weak hysteresis type.”

According to this definition, we speculate that the traits of Type-2 cancer cells as: 1) TME-driven EMT: The driving force of EMT originates from the TME. 2) Sustained motile mesenchymal state: These cancer cells can be maintained in a motile mesenchymal state. 3) Spontaneous MET process: The MET process is spontaneous as cancer cells leave the TME and withdraw from EMT-inducing factors. 4) Regained epithelial phenotype: After MET, cancer cells regain the epithelial phenotype, restoring their proliferative ability and initiating colonization for metastasis. 5) Formation of new TME: Successful colonization and formation of metastatic foci result in the establishment of a new TME, where new TILs can be recruited. The EMT-inducing factors signals from these TILs may induce a new round of “EMT-MET-colonization” process, leading to further tumor spread. 6) Short-lived mesenchymal phenotype: The mesenchymal phenotype, while a major form of cell migration, apoptosis resistance, and chemoresistance, is of low efficacy due to the brief duration of the mesenchymal state. The aggressiveness of Type-2 cancer cells was stronger than that of Type-1 but weaker than that of Types 2 and 3.

### 5.3 Type-3 (strong hysteresis type)

While Type-3 cancer cells are very similar to Type-2, they have stronger hysteresis than Type-2 cancer cells. This allows Type-3 cancer cells to remain in a mesenchymal state for longer periods, with longer migration distance, longer apoptosis-resistance time, and greater possibility for successful metastasis than their Type-2 counterparts. Additionally, the plasticity of Type-3 was greater than that of Type-2, indicating the greater aggressiveness of Type-3 than that of Types 1 and 2.

For Type-3 cancer cells, the strong hysteresis makes the MET process a slow process and may emerge multiple intermediate states between the complete mesenchymal and epithelial states, which indicates that some cancer cells may experience a transient “hybrid epithelial/mesenchymal phenotype,” leading to enhanced tumor aggressiveness. In addition to the transient hybrid state, desynchronization of the MET process among migrating cancer cells endows these cells with variant levels of E-cadherin and N-cadherin, which may result in the collective cluster migration of cancer cells.

### 5.4 Type-4 (stable hybrid epithelial/mesenchymal type)

Multiple studies have demonstrated the existence of a stable hybrid epithelial-mesenchymal phenotype. Cancer cells of this type simultaneously possess the traits of epithelial and mesenchymal cells and have the highest plasticity and aggressiveness. These cancer cells are proliferative, migratory, and chemo-resistant, often culminating in the terminal stage of tumors. Hybrid epithelial and mesenchymal cancer cells collectively migrate (Jolly et al., 2016). Type-4 cancer cells are relatively few and can only be seen in substantial and rapidly progressing malignancies and in treatment-resistant terminal cases.

### 5.5 Molecular characteristics of the four types

The formation of these four types is complicated and is not determined by a single factor. Therefore, it was not feasible to delineate the specific molecular background of each type. Generally, for Type-1, the EMT-promoting genes are supposed to be overexpressed. These genes are thought to drive EMT by directly acting on EMT-TFs but not by affecting the microenvironment. Type-2 and Type-3 are thought to have more epigenetic modifications that involve EMT memory than Type-2. Phenotypic stability factors in Type-4 are thought to be overexpressed.

### 5.6 Comparison of the four types

The cancer cells with EMP potential were classified into four groups with varying biologies. While the EMT in Type-1 and Type-4 are generally induced by internal factors, Type-2 and Type-3 are TME-dependent. Plasticity and aggressiveness are Type-1 weaker than in Type-2 and weaker in Type-3 than in Type-4.

## 6 Clinical hypotheses based on the classification theory (with colorectal cancer example)

### 6.1 Oligometastasis, metachronous metastasis, and tumor dormancy

Oligometastatic disease has been proposed as an intermediate state between localized and systemic metastatic disease (Hellman and Weichselbaum, 1995). Patients with oligometastasis are reported to have better overall survival than those without oligometastasis after treatment (Ruers et al., 2017; Gomez et al., 2019). However, little progress has been made in understanding and defining oligometastatic diseases based on tumor biology. Synchronous metastatic disease is associated with a more aggressive disease phenotype and worse prognosis than metachronous metastatic disease (Fong et al., 1999; Tsai et al., 2007; Ashworth et al., 2014). The mechanism by which metachronous metastasis is superior to synchronous metastasis at the biological level remains unclear. In this study, we attempted to interpret these phenomena based on our classification theory. For Type-1, cancer cells first undergo an EMT process, transition to a complete mesenchymal state and then acquire migratory ability. In the complete mesenchymal state, these cancer cells lack adhesive ability and migrate as single cells instead of as tumor clusters. When these single cancer cells arrive at the target organ, they cannot transition to the mesenchymal state and lack proliferative ability. Therefore, these single cancer cells remain silent within the target organs without proliferating and cannot initiate colonization and become “tumor dormant.” After a certain period, noise, oscillations inside cancer cells, or MET-stimulating signals from the TME may appear stochastically and induce the MET process. These dormant cancer cells can transition to an epithelial state, regain their proliferative ability, initiate colonization, and cause distal metastasis. As MET is a stochastic event, it has low efficacy (fewer metastatic lesions) and requires time to occur (metachronous), expressing an oligometastatic and metachronous metastatic pattern. As discussed previously, Type-2 cancer cells have low efficacy for distal migration. Type-3 cancer cells are more likely to cause multiple metastases to target organs. Type-4 cancer cells are more likely to metastasize to multiple target organs. Therefore, we speculate that “oligometastasis” and “metachronous metastasis” are the unique traits of Type-1 cancer cells. Type-1 cancer cells are thought to be less aggressive than the other types owing to their weak EMP. Therefore, we hypothesized that oligometastatic and metachronous metastasis predict better prognosis than non-oligometastatic and synchronous metastasis, which can contribute to the fact that cancer cells in metachronous metastasis and oligometastasis are Type-1 cancer cells, which have low EMP and less aggressive biology.

### 6.2 Tumor border, tumor budding, and extramural non-nodal tumor deposits

Tumor borders, budding, and extramural non-nodal tumor deposits have prognostic significance. The prognostic significance of the tumor border was independent of tumor-node-metastasis staging. An irregular infiltrating growth pattern rather than a smooth “pushing” (expansile) border is considered an independent adverse prognostic factor (Morikawa et al., 2012; Abe et al., 2022). Tumor budding is defined as microscopic clusters of undifferentiated cancer cells at the invasive tumor front (Hase et al., 1993; Oh et al., 2018). Extensive tumor budding may have a better prognostic value than tumor grade, which is independent of the overall tumor border configuration (Oh et al., 2018). Extramural non-nodal tumor deposits are defined as “discrete tumor nodules within the lymph drainage area of the primary carcinoma without identifiable lymph node tissue or identifiable vascular or neural structure.” The presence of tumor deposits is a strong adverse prognostic feature (Lord et al., 2017; Nagtegaal et al., 2017). Tumor deposits along with lymph node metastases, are the strongest predictors of liver and peritoneal metastases (Nagtegaal et al., 2017). Tumor deposits are not equivalent to lymph node metastases concerning biology and outcomes. The biology of the relationship between adverse prognoses and tumor borders, tumor budding, and extramural non-nodal tumor deposits is unknown.

Here, we attempt to interpret biology based on the classification theory. Cancer cells of Type-1 are in the mesenchymal state, lack E-cadherin, and cannot adhere to form clusters, moving individually (Jolly et al., 2016). These single mesenchymal cancer cells can migrate out of primary cancer lesions. As the cancer cells of Type-1 can migrate a long distance away from the primary lesion and are kept in single-cell forms instead of clusters, they do not cause an irregular tumor, and there are no tumor buddings or tumor deposits around the primary lesion. For Type-2 cancer cells, mesenchymal cancer cells can migrate out of the primary cancer lesion, and due to weak hysteresis, as soon as cancer cells leave the TME, they will transition back to the epithelial state immediately and then reacquire adhesive and proliferative abilities, which would lead to cluster emergence. Therefore, Type-2 cancer cells can lead to irregular tumor borders and budding. For Type-3 cancer cells with strong hysteresis, mesenchymal cancer cells can migrate a greater distance within the colorectal mesentery than Type-2. Therefore, in addition to the primary tumor, cancer cells can also be observed in the mesentery, which is far from the primary cancer lesion. When these cancer cells of Type-3 transition to the epithelial state, they transiently undergo a hybrid epithelial/mesenchymal state and gain adhesive and proliferative abilities. Therefore, they can adhere to and proliferate to form cancer clusters or nodes, resulting in detectable tumor deposits. Type-4 cancer cells have strong migratory and proliferative abilities. These cancer cells can collectively migrate from the primary lesion, leading to irregular tumor borders and budding. Further, they collectively migrate in the mesentery as clusters and even proliferate during the migration process to enlarge the cluster and form extramural non-nodal tumor deposits.

Therefore, we proposed the following hypotheses: 1) Tumor border, tumor budding, and extramural non-nodal tumor deposits have prognostic significance because they can reflect the classification of cancer cells. A smooth tumor indicates Type-1 cancer cells or no EMT-potential cancer cells in the primary tumor. Irregular tumor borders and only budding (no tumor deposits) indicate Type-2 cancer cells. The coexistence of irregular tumor borders, budding, and deposits indicates Type-3 and Type-4 cancer cells. 2) Previously, tumor deposits were considered lymph nodes, in which the lymphatic structure was replaced by cancer cells. According to our theory, mesenteric tumor deposits are formed by the adhesion and proliferation of Type-3 or Type-4 cancer cells. 3) It has been reported that tumor deposits in combination with lymph node metastases are the strongest predictor for liver and peritoneal metastases (Nagtegaal et al., 2017). Co-existing lymph node metastasis and tumor deposits indicate the presence of Type-3 cancer cells, and the presence of Type-3 cancer cells indicates strong aggressiveness of the tumor and is more likely to cause tumor spreading.

### 6.3 Considerations in regional lymph nodes metastasis

For colorectal cancer, regional lymph node involvement was the strongest predictor of outcomes after surgical resection of colorectal cancer, second only to the presence of distant metastases. Occult tumor cells can be categorized as micrometastases (MMs) or isolated tumor cells (ITCs). Isolated tumor cells (ITCs) in colorectal cancer are defined as single cancer cells or small clusters of tumor cells measuring ≤0.2 mm (Jin and Frankel, 2018). However, MMs are defined as clusters of tumor cells measuring ≥0.2 mm in greatest dimension. In patients with colorectal cancer, disease recurrence was considerably increased in the presence of MMs in regional lymph nodes compared to that in the absence of occult tumor cells, whereas disease recurrence did not increase in the presence of ITCs in regional lymph nodes (Sloothaak et al., 2014). Isolated tumor cells do not have predictive value (Sloothaak et al., 2014). Regional lymph node involvement in MMs and ITCs has variable prognostic value. According to our results, ITCs are Type-1 cancer cells in a mesenchymal state and lack adhesive capacity. These Type-1 cancer cells move in single-cell forms and cannot gather to form larger clusters. Hence, they are categorized as “ITCs.” As discussed previously, Type-1 cancer cells have a relatively good prognosis. Therefore, ITCs are not indicators of poor prognosis. ITCs are single migratory cancer cells found in the lymph nodes. These cancer cells are in a complete mesenchymal state; therefore, they maintain a single-cell migration pattern and are not indicators of poor prognosis, which explains why ITCs do not demonstrate poor prognosis. Further, MMs are Type-3 cancer cells with strong hysteresis. As Type-3 cancer cells migrate out of the TME in primary tumor lesions, they gradually transition to an epithelial state. In the MET process, cancer cells experience a short-term hybrid epithelial/mesenchymal state and can adhere to form MMS or proliferate to form MMS instead of ITCs. As discussed above, colorectal cancer with Type-2 cancer cells has a worse prognosis than colorectal cancer with Type-1 cancer cells. The presence of MMS or ITCs, but not visible lymph node metastasis, can exclude the presence of Type-4 cancer cells because Type-4 cancer cells would cause widespread lymph node metastasis instead of occult tumor cells. Taken together, we speculate that the prognostic variation between MMS and ITCs in regional lymph nodes is indeed a variance in cancer cell types.

In colorectal cancer, the lymph node ratio (LNR; the ratio of metastatic lymph nodes to the total number of examined lymph nodes) is an independent predictor of overall, disease-free, and cancer-specific survival, and the prognostic separation obtained by the LNR is superior to that of the number of positive nodes (Swanson et al., 2003; Johnson et al., 2006). Although the underlying mechanisms are not entirely clear, studies have shown that the number of normal lymph nodes retrieved from resected specimens conveys important prognostic information for both stage II and III colon cancer (Swanson et al., 2003; Johnson et al., 2006). The reason for the relationship between the total number of nodes in the specimen and outcomes is unclear. The most obvious explanation is that removing more nodes increases staging accuracy. However, the strong association between the total lymph node count and survival is not entirely explained by improvements in staging (Baxter et al., 2010; Moore et al., 2010; Parsons et al., 2011). To interpret this phenomenon according to our theory, when Type-1 cancer cells migrate through regional lymphatic systems, tumor factors secreted by migrating cancer cells may stimulate lymph node enlargement. However, these single cancer cells (non-ITCs) generally cannot be identified if additional special/ancillary techniques such as pan-cytokeratin immunohistochemical staining (Estrada et al., 2017) are used. This scenario is clinically considered as negative lymph nodes. Therefore, enlarged but negative lymph nodes would decrease the LNR. According to our theory, enlarged but negative lymph nodes imply the presence of Type-1 cancer cells instead of Type-3 or Type-2 cancer cells. Therefore, the intrinsic characteristic of low LNR that predicts a good prognosis is that low LNR demonstrates the presence of Type-1 cancer cells instead of Type-3 or Type-4 cancer cells.

Here, we propose a method to verify our theory and speculation. As discussed in this section, ITCs and low LNRs are unique traits of Type-1 cancer cells. Therefore, we hypothesized that in colorectal cancer patients, ITCs and low LNRs are more likely to exist simultaneously and that these patients (with ITCs and low LNR) are more likely to undergo oligometastasis and metachronous metastasis than non-oligometastasis and synchronous metastasis.

### 6.4 Peritoneal metastasis

Metastases occur in the peritoneum in 25% of the patients and result in a poorer prognosis compared with other sites of metastasis in colorectal cancer (Segelman et al., 2012). Peritoneal metastasis is a distal metastasis and is classified as terminal-stage cancer. In a combined study of 2095 patients with metastatic colorectal cancer, peritoneal carcinomatosis was present in 364 patients (17%), and only 44 (2.1%) displayed peritoneal carcinomatosis as the sole manifestation of metastatic disease (Segelman et al., 2012). Some patients with peritoneal metastases achieve long-term survival after aggressive cytoreduction surgery with hyperthermic intraperitoneal chemotherapy (Breuer et al., 2021).

In the present study, we attempted to interpret these prognostic variants. We speculate the following: 1) As discussed previously, Type-1 cancer cells can migrate to the peritoneum as their target organ. Later, after stochastic MET, they initiate colonization and form metastatic foci. This kind of peritoneal metastasis is supposed to be single-located, and surgical resection of this kind of metastasis could achieve a relatively good prognosis. 2) In Type-2 or Type-3 cells, mesenchymal cancer cells can directly migrate from the primary lesion or mesentery and arrive at the adjacent peritoneum. After they arrival in the peritoneum, MET spontaneously occurs, transitions back to the epithelial state, and proliferates to form metastatic foci. The variance between Type-2 and Type-3 is that Type-3 could migrate a greater distance as Type-3 maintained a longer time in the mesenchymal state due to strong hysteresis. The extent of Type-3 is wider than Type-2. For Type-2 cancer cells, peritoneal metastasis is more likely to be “a locally advanced tumor” rather than a distal metastasis. In this scenario, peritoneal metastasis is resectable with a relatively favorable prognosis. For either Type-2 or Type-3, if the primary peritoneal metastatic lesion is left alone, the cancer cells in the peritoneal metastatic lesion will proliferate and enlarge. If the size is big enough, they can build their own TME, recruit TILs, secrete EMT-inducing factors, such as TGF-β, and promote new rounds of “EMT-MET-colonization” processes, cause cancer cells to spread continuously, and ultimately to a widespread peritoneal metastasis pattern. 3) For Type-4, hybrid state cancer cells possess strong mobility and growth capabilities. If cancer cells arrive in the peritoneum, they rapidly spread and are accompanied by metastases to other organs.

## 7 Conclusion

In this study, based on EMP, EMT hysteresis, and the hybrid epithelial/mesenchymal phenotype, we classified cancer cells with EMP potential into four types and subsequently elucidated the spreading patterns of these types of cancer cells. In the hypotheses section, we attempted to interpret the corresponding associations between the types and clinical phenomena. This classification system provides a framework to elucidate variations in cancer cell spreading patterns and behavior during metastasis. It allows for predicting the future movement of cancer cells, informing tailored treatment strategies. This system’s novelty lies in its specific focus on cancer cells with EMP potential, a significant contributor to prognosis, rather than the entire tumor. Multiple types (here “types” refers types in our classification system) of cancer cells coexist within the same tumor, making tumor spreading pattern complicated. Further studies are needed to validate the classification system at both preclinical and clinical levels.

## Data Availability

The original contributions presented in the study are included in the article/supplementary material, further inquiries can be directed to the corresponding author.
